# Effect of acute kidney injury requiring extended dialysis on 28 day and 1 year survival of patients undergoing interventional lung assist membrane ventilator treatment

**DOI:** 10.1186/1471-2369-12-15

**Published:** 2011-04-13

**Authors:** Jan T Kielstein, Soeren Tolk, Carsten Hafer, Anna Heiden, Olaf Wiesner, Christian Kühn, Johannes Hadem, Marius M Hoeper, Stefan Fischer

**Affiliations:** 1 Department Nephrology and Hypertension, Medical School Hannover, Germany; 2 Department of Pulmonary Medicine, Medical School Hannover, Germany; 3 Department of Cardiothoracic Surgery, Medical School Hannover, Germany; 4 Department of Gastroenterology, Hepatology and Endocrinology, Medical School Hannover, Germany

## Abstract

**Background:**

Extracorporeal lung assist devices are increasingly used in the intensive care unit setting to improve extracorporeal gas exchange mainly in patients with acute respiratory distress syndrome. ARDS is frequently accompanied by acute kidney injury; however it is so far unknown how the combination of these two conditions affects long term survival of critically ill patients.

**Methods:**

In a retrospective analysis of a tertiary care hospital we evaluated all patients undergoing interventional lung assist (iLA) treatment between January 1^st ^2005 and December 31^st ^2009. Data from all 61 patients (31 F/30 M), median age 40 (28 to 52) years were obtained by chart review. Follow up data up to one year were obtained.

**Results:**

Of the 61 patients undergoing iLA membrane ventilator treatment 21 patients had acute kidney injury network (AKIN) stage 3 and were treated by extended dialysis (ED). Twenty-eight day survival of all patients was 33%. While patients without ED showed a 28 day survival of 40%, the survival of patients with ED was only 19%. Patients on ED were not different in respect to age, weight, Horowitz index and underlying disease.

**Conclusions:**

AKI requiring ED therapy in patients undergoing iLA treatment increases mortality in ICU patients. Patients in whom iLA was placed as a bridge to lung transplantation and that were successfully transplanted showed the best outcome. Future studies have to clarify whether it is possible to identify patients that truly benefit from the combination of these two extracorporeal treatment methods.

## Background

The most frequent contributing factor to acute kidney injury (AKI) is sepsis and multi-organ dysfunction syndrome (MODS) [1]. The high mortality rate of patients with AKI reaches 60% despite considerable improvement of renal replacement therapy (RRT). This fostered the interest on the impact of AKI on distant organ function. One interesting interaction is the one between lung and kidney. AKI induces increased lung vascular permeability, cellular inflammation, and dysregulated salt and water channels resulting in respiratory failure [2]. Also in patients with non-severe pneumonia AKI is associated with higher immune response and an increased risk of death [3]. In contrast to several studies investigating the effect of AKI on survival of patients undergoing extracorporeal membrane oxygenation [4,5], there is only scarce data on the effect of AKI on the survival of patients undergoing pumpless interventional lung assist (iLA membrane ventilator; Novalung, Talheim, Germany) treatment. This technique is increasingly used by intensivists in patients with life-threatening respiratory failure or acute respiratory distress syndrome (ARDS) suffering from persistent hypercapnia. The 28 day survival of patients undergoing iLA therapy ranges from 41% in a retrospective analysis [6] to 51% in a prospective cohort study in patients with ARDS from multiple etiologies [7]. However, those studies disregarded patients with AKI on renal replacement therapy (RRT). Only two studies provide short term survival data on patients undergoing iLA membrane ventilator therapy also suffering from AKI. In a retrospective analysis by Liebold et al. 39% of the iLA patients required intermittent haemodialysis [8]. In a study by Floerchinger et al. 42% of the patients were treated by continuous veno-venous hemofiltration (CVVH) [9]. So far it is unknown, what impact extended dialysis (ED), an increasingly used RRT method in the ICU [10,11], has on long term survival of patients undergoing iLA treatment. Moreover, it is not known whether the improvement in respiratory acidosis has any impact on renal function in these patients. Hence, the aim of our study was to investigate the effect of AKI on long term mortality of patients treated by iLA. Furthermore the potential impact of iLA treatment on renal function was investigated in this single centre retrospective study.

## Methods

This retrospective cohort study included all adult patients that underwent iLA treatment from Januar 1st 2005 to December 31st 2009 at a tertiary care university hospital. The Institutional Review Board of the Medical School Hannover waived the need for approval and informed consent for this analysis. A total of 61 patients were identified in the central documentation system of the hospital. From all of those patients charts as well as laboratory data were reviewed. RRT dependence was defined as AKIN stage 3. All patients requiring RRT in addition to iLA treatment received ED using the GENIUS system. Details of the system are summarized elsewhere [11].

Estimated glomerular filtration rate (eGFR) was calculated using the CKD-EPI formula [12].

### Statistical analysis

We used GraphPad Prism 5 for statistical analysis. ANOVA was used to compare patients requiring extended dialysis to patients undergoing iLA therapy only. Kaplan-Meier plots were analyzed for significance using the Kruskal-Wallis test. The significance level was set at p < 0.05.

## Results

**Figure 1:**
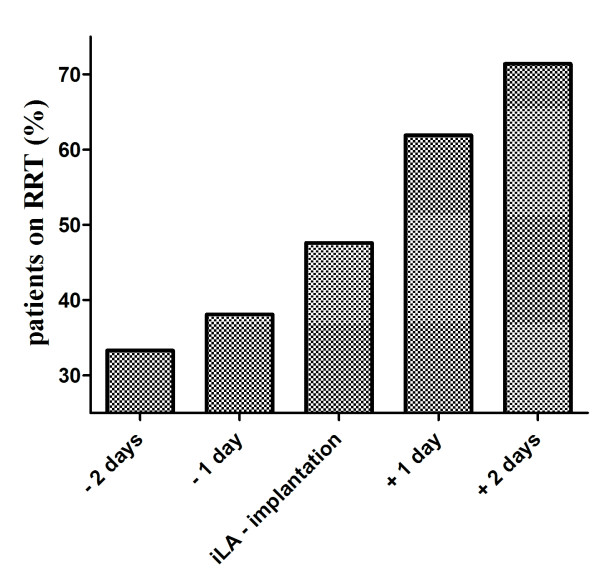
**Depicts the percentage of RRT (renal replacement therapy) dependent patients in relation to the iLA-implantation**. Refers to RRT dependent patients (n = 21) only.

**Figure 2:**
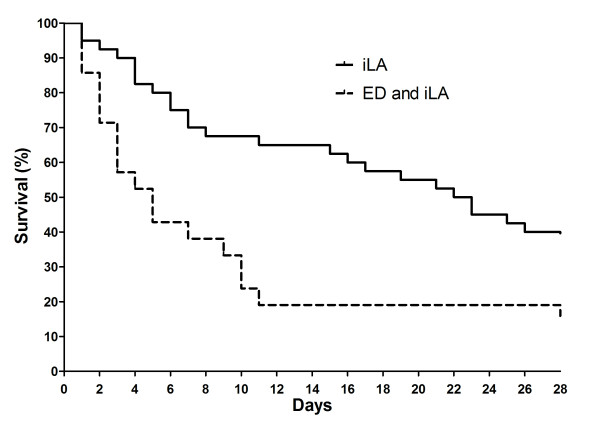
**Kaplan Meier survival curve of patients undergoing iLA treatment only (n = 40) or the combination of iLA treatment and ED (extended dialysis) (n = 21)**.

**Figure 3:**
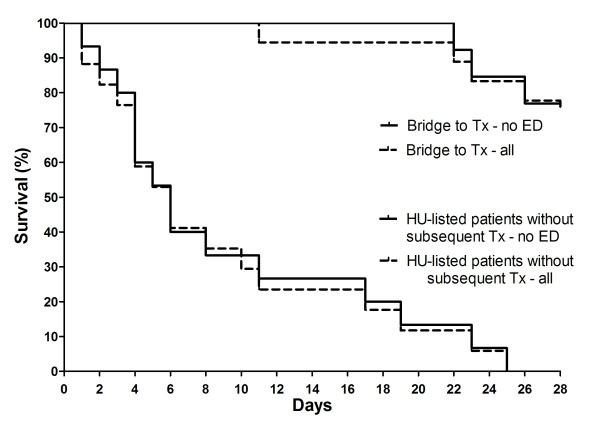
**Kaplan-Meier survival curves of patients successfully bridged to lung transplantation (n = 18) or patients with HU (high urgency) status not being transplanted (n = 17)**. Survival in both groups was not affected by the need for ED (extended dialysis).

We identified 61 patients undergoing iLA treatment within our observation period of five years. Patient characteristics are presented in Table 1. The underlying diseases are summarized in Table 2. More than one third (n = 21, 34%) of all patients required ED. Exactly one third of these patients had already been RRT dependent before (n = 7; 33%) iLA insertion. Half of the patients became RRT dependent on the day of iLA insertion (n = 10; 48%). Within two days after iLA placement another five patients (24%) became RRT dependent. Hence, within two days of iLA insertion 71% (n = 15) of patients ever to become RRT dependent were on ED (Figure 1). This corresponds to roughly 25% of all patients undergoing iLA treatment. Patients undergoing ED treatment were not different in respect to gender, age, weight, iLA support duration, use of nephrotoxic antiobiotics, PaO_2_, paCO_2_, Horowitz index, MAP, P_max _and PEEP at start of iLA support (Table 1) and underlying disease to patients not requiring RRT. Also time from ICU admission to iLA insertion was not different in the two groups. However, patients undergoing ED had a significant lower pH, a higher SOFA and APACHE II score as well as a significant higher iLA flow 2 h after implantation than patients not requiring RRT (p < 0.05) (Table 1). The 28 day survival of the whole cohort was 23%. There was a clear difference in survival between patients requiring ED and those not requiring RRT. While patients undergoing ED exhibited a 19% 28 day survival, the survival rate at day 28 in patients without need for dialysis was 40% (p = 0.001) (Figure 2). This difference became even more marked after one year. While patients undergoing ED had a 5% one year survival, patients without the need for RRT had a 25% one year survival (p < 0.001). Patients in whom iLA was placed as a bridge to lung transplantation and that were successfully transplanted showed a markedly higher 28 day survival rate of 78%, while all of the patients listed for lung transplantation without subsequently receiving a graft died before day 25 (Figure 3). In contrast to the whole cohort, the necessity of RRT had no effect on survival in the transplant candidates.

**Table 1:** Patient characteristics before iLA insertion (unless otherwise stated).

	all	iLA and ED	iLA - no ED	p
patients	61	21	40	
male/female	30 | 31	9 | 12	21 | 19	
age (years)	40 (28 to 52)	36 (28 to 52)	41 (28 to 49)	0.942
iLA treatment (days)	6 (3 to 11)	5 (2 to 9)	7 (5 to 13)	0.191
iLA-flow after 2 h (l/min)	1.1 (1.0 to 1.4)	1.4 (1.3 to 1.6)	1.1 (1.0 to 1.1)	**0.009**
days on ICU until iLA	7 (3 to 15)	8 (5 to 16)	7 (3 to 12)	0.880

paO_2 _(mmHg)	82 (70 to 98)	77 (70 to 96)	82 (70 to 100)	0.97
after 24 h iLA	68 (65 to 84)	71 (62 to 90)	78 (68 to 83)	0.900
paCO_2 _(mmHg)	100 (81 to 115)	98 (81 to 114)	100 (79 to 115)	0.880
after 24 h iLA	52 (43 to 61)	44 (53 to 59)	56 (45 to 61)	0.840
pH	7.15 (7.08 to 7.26)	7.10 (7.04 to 7.15)	7.23 (7.13 to 7.27)	**0.007**
after 24 h iLA	7.38 (7.31 to 7.46)	7.34 (7.26 to 7.35)	7.41 (7.33 to 7.46)	**0.013**
Horowitz index (PaO_2_/FiO_2)_	96 (75 to 137)	98 (77 to 114)	94 (67 to 144)	0.280
MAP (mmHg)	80 (75 to 86)	80 (70 to 85)	80 (78 to 87)	0.249
P_max _(cmH_2_O)	38 (34 to 41)	38 (35 to 40)	39 (32 to 42)	0.747
PEEP (cmH_2_O)	8 (5 to 10)	9 (5 to 13)	8 (5 to 10)	0.370
Creatinine* (μmol/l)	53 (40 to 91)	93 (49 to 174)	50 (37 to 73)	**0.005**
after 24 h iLA			52 (34 to 78)	
eGFR* (ml/min/1,73 m^2^)	120 (79 to 138)	66 (33 to 138)	124 (100 to 140)	**0.003**
SOFA-Score	11 (10 to 12)	14 (11 to 17)	11 (10 to 11)	**< 0.0001**
APACHE II - Score	32 (28 to 34)	32 (31 to 38)	31 (28 to 33)	**0.023**

Vancomycin use day 1-3 of iLA	32%	40%	29%	
Aminoglycoside use day 1-3 of iLA	26%	13%	32%	

28 day survival	33%	19%	40%	
one year survival	18%	5%	25%	

Data presented as median (interquartile ranges). *Creatinine and eGFR only from patients without ED dependence before iLA insertion.

**Table 2:** Underlying disease and survival at 28 days and one year.

underlying disease	n	28 day survival	one year survival
cystic fibrosis	15	27%	27%
pneumonia	10	40%	20%
status post lung Tx	8	25%	13%
pulmonary fibrosis	7	43%	0%
COPD	7	29%	0%
lung cancer	2	50%	0%
pulmonary hypertension	2	100%	50%
leukemia	2	50%	50%
polytrauma	2	50%	50%
others	6	17%	17%

Not surprisingly, the median eGFR of patients with no current or future need of ED on admission to the ICU was 124 (100 to 140) [ml/min/1.73 m^2^]. Patients which became RRT dependent had a significant lower eGFR (66 (33 to 138) [ml/min/1.73 m^2^]). Despite the improvement in pH and pCO_2 _the insertion of iLA had no effect on renal function in patients that did not become RRT dependent (Table 1).

## Discussion

The pertinent findings of our study were that 1) the combination of iLA treatment and ED is associated with an increased short and long term mortality 2) most of the patients with this combination either were dialysis dependent before the start of iLA treatment or within 48 hours after start of iLA therapy 3) the bridging to successful lung transplantation was associated with the best 28 day survival 4) decreased eGFR at the time on the day of iLA institution was predictive of future dialysis dependence.

### iLA treatment and RRT

This report is the first evaluation of the influence of ED on the survival of patients undergoing iLA treatment. Two previous studies on iLA treatment did not address this issue of AKI and renal replacement therapy at all [6,7]. The rate of patients requiring renal replacement therapy in our cohort (34%) is comparable with two previous retrospective studies in which the rate AKI requiring renal replacement therapy was 39 and 42% [8,9]. These studies also investigated the influence of either intermittent hemodialysis [8] or CVVH [9] on survival. In the retrospective analysis by Liebold and co-workers 39% of all patients required intermittent hemodialysis [8]. These authors reported also a low survival rate of 15% (4 out of 27 patients), in patients requiring both dialysis and iLA, while 21 of 43 (49%) of those patients not requiring dialysis survived. In the study by Floerchinger et al. 42% of the patients required CVVH. The survival rate of those patients was 19%. The fact that outcome is worse in a given acutely ill patient population, if AKI necessitating RRT develops, is of course not exceptional and has been depicted in many populations (for review see [13]). Indeed, patients that needed both iLA and ED were sicker as reflected by disease severety scores, a difference however mainly caused by the deteriorating renal function. Interestingly, the use of nephrotoxic antibiotics in both groups was not different. Still, the exceptionally high mortality of 81% in patients suffering from (temporary) two organ failure in our analysis is remarkable as the average hospital mortality in ICU patients requiring RRT was 60% in a large multinational study [1]. The effect of RRT on survival was not detectable in patients who survived until lung transplantation, indicating that prognosis of critically ill patients improves if one organ system can be substantially improved.

### Effect of iLA on renal function

Preclinical data suggested that respiratory acidosis induces a drop in GFR, effective renal plasma flow and urine output [14]. Accordingly we assumed that amelioration of respiratory acidosis would improve renal function. However, for those patients not undergoing RRT serum creatinine did not significantly change over the period of iLA support.

We wish to point out limitations of our study. Firstly the retrospective design and the single centre setting are important limitations, yet we included patients from different departments, i.e. surgical and medicine, into the analysis. Also the number of patients is limited, however our 21 patients with iLA and ED treatment significantly add to the 27 and 67 patients treated by iLA and RRT published previously [8,9]. Moreover our study provides a one year follow up of the patients, which has so far not been reported by any other group investigating the effect of the combination of RRT and iLA treatment. Another important limitation is the fact that we included a heterogeneous set of patients in which 36% were either status post transplantation or underwent lung transplantation after iLA treatment. Last but not least we did exclude patients on extracorporeal membrane oxygenation

## Conclusion

The combination of iLA treatment with acute kidney injury requiring renal replacement therapy dramatically increases mortality. Most of the patients that become dialysis dependent do so within 48 hours of iLA insertion. These patients have already a decreased eGFR at the time of ICU admission. The best outcome in our cohort could be obtained in patients that were successfully bridged to transplantation.

## Abbreviations

AKI: acute kidney injury; CVVH: continuous veno-venous hemofiltration; ED: extended dialysis; GFR: glomerular filtration rate; iLA: interventional lung assist

## Competing interests

The authors declare that they have no competing interests.

## Authors' contributions

JTK, ST and SF designed the study. ST, CH, AH, OW and CK were involved in the data acquisition. JTK and ST analyzed the data. JTK, ST, JH and MMH contributed to the interpretation of the data and manuscript drafting. All other authors reviewed the manuscript. All authors read and approved the final manuscript.

## Pre-publication history

The pre-publication history for this paper can be accessed here:

http://www.biomedcentral.com/1471-2369/12/15/prepub
